# Diesel Particulate Matter 2.5 Induces Epithelial-to-Mesenchymal Transition and Upregulation of SARS-CoV-2 Receptor during Human Pluripotent Stem Cell-Derived Alveolar Organoid Development

**DOI:** 10.3390/ijerph17228410

**Published:** 2020-11-13

**Authors:** Jung-Hyun Kim, Jeeyoung Kim, Woo Jin Kim, Yung Hyun Choi, Se-Ran Yang, Seok-Ho Hong

**Affiliations:** 1 Department of Internal Medicine, School of Medicine, Kangwon National University, 1 Kangwondaehakgil, Chuncheon 24341, Korea; katop2024@naver.com (J.-H.K.); jeeyoung0628@gmail.com (J.K.); pulmo2@kangwon.ac.kr (W.J.K.); 2 Environmental Health Center, Kangwon National University Hospital, Chuncheon 24341, Korea; 3 Department of Biochemistry, College of Korean Medicine, Dong-eui University, Busan 47227, Korea; choiyh@deu.ac.kr; 4 Department of Thoracic and Cardiology, School of Medicine, Kangwon National University, 1 Kangwondaehakgil, Chuncheon 24341, Korea; seran@kangwon.ac.kr

**Keywords:** dPM2.5, EMT, alveolar organoid, human pluripotent stem cells, fibrosis

## Abstract

Growing evidence links prenatal exposure to particulate matter (PM2.5) with reduced lung function and incidence of pulmonary diseases in infancy and childhood. However, the underlying biological mechanisms of how prenatal PM2.5 exposure affects the lungs are incompletely understood, which explains the lack of an ideal in vitro lung development model. Human pluripotent stem cells (hPSCs) have been successfully employed for in vitro developmental toxicity evaluations due to their unique ability to differentiate into any type of cell in the body. In this study, we investigated the developmental toxicity of diesel fine PM (dPM2.5) exposure during hPSC-derived alveolar epithelial cell (AEC) differentiation and three-dimensional (3D) multicellular alveolar organoid (AO) development. We found that dPM2.5 (50 and 100 μg/mL) treatment disturbed the AEC differentiation, accompanied by upregulation of nicotinamide adenine dinucleotide phosphate oxidases and inflammation. Exposure to dPM2.5 also promoted epithelial-to-mesenchymal transition during AEC and AO development via activation of extracellular signal-regulated kinase signaling, while dPM2.5 had no effect on surfactant protein C expression in hPSC-derived AECs. Notably, we provided evidence, for the first time, that angiotensin-converting enzyme 2, a receptor to mediate the severe acute respiratory syndrome coronavirus clade 2 (SARS-CoV-2) entry into target cells, and the cofactor transmembrane protease serine 2 were significantly upregulated in both hPSC-AECs and AOs treated with dPM2.5. In conclusion, we demonstrated the potential alveolar development toxicity and the increase of SARS-Cov-2 susceptibility of PM2.5. Our findings suggest that an hPSC-based 2D and 3D alveolar induction system could be a useful in vitro platform for evaluating the adverse effects of environmental toxins and for virus research.

## 1. Introduction

Particulate matter (PM), as a major component of air pollutant, is known to elevate susceptibility to pulmonary diseases, including asthma, chronic obstructive pulmonary disease, fibrosis, and lung cancer [1]. Evidence from epidemiologic studies links prenatal exposure to fine particulate matter (PM2.5) with adverse respiratory outcomes in infancy and childhood. A few earlier epidemiologic studies have shown the potential associations of in utero PM exposure with an increased risk of developing asthma in children [2,3]. Recent epidemiologic studies with a larger birth cohort demonstrated that higher prenatal PM2.5 exposure at mid-gestation was associated with an increased incidence of childhood asthma [4,5] and significant deficits in lung function in early childhood [6,7]. Findings from biological and genetic studies indicate that prenatal exposure to PM2.5 induces oxidative stress, inflammation, and epigenetic modifications, which may contribute to adverse respiratory outcomes in childhood [8]. A recent animal study demonstrated that maternal exposure to PM2.5 resulted in lung inflammation in offspring, which is mediated by upregulation of high-mobility group box-1 [9]. However, the underlying cellular and molecular mechanisms for these pathologies are not fully understood, which might explain the lack of an ideal in vitro human lung developmental model.

Human pluripotent stem cells (hPSCs) offer an invaluable resource for evaluating in vitro developmental toxicity due to their unique ability to differentiate into any type of cell in the body. In fact, hPSC-derived functional derivatives including cardiomyocytes, keratinocytes, and fibroblasts have been successfully employed for developmental toxicity evaluations of PM [10,11,12]. However, two-dimensional (2D) cell culture systems used in these studies are limited in recapitulating the complexity and functions of in vivo tissues and may provide inaccurate cellular and molecular responses to environment toxin exposure. Thus, the need for a more accurate model system has triggered the development of three-dimensional (3D) structures from hPSCs, which are able to circumvent the rare accessibility to primary human tissues as well as more faithfully recapitulate the native organ [13]. In the lungs, several types of organoids that represent different respiratory compartments such as proximal and distal airways have been successfully generated from hPSCs and have already proven to be an excellent platform for understanding the molecular mechanisms of early lung development and modeling pulmonary diseases [14,15,16,17]. However, to date, no studies have been conducted to investigate the adverse effects of PM2.5 exposure during hPSC-derived 2D and 3D alveolar development.

In order to investigate the adverse effects of diesel fine PM (dPM2.5) during alveolar development, we employed an optimized stepwise induction protocol to generate functional alveolar epithelial cells (AECs) and 3D multicellular alveolar organoids (AOs) from hPSCs. We demonstrated that dPM2.5 disturbed the AEC differentiation and promoted epithelial-to-mesenchymal transition (EMT) during alveolar development via activation of extracellular signal-regulated kinase (ERK) pathway. We also provided evidence, for the first time, that dPM2.5 treatment during alveolar development can upregulate angiotensin-converting enzyme 2 (ACE2) as the receptor for the severe acute respiratory syndrome coronavirus clade 2 (SARS-CoV-2) and its cofactor transmembrane serine protease 2 (TMPRSS2).

## 2. Materials and Methods

### 2.1. Cell Cultures

The human adenocarcinoma lung epithelial type II cell line A549 was cultured in Dulbecco’s Modified Eagle Medium with 10% fetal bovine serum (FBS) and 1% Penicillin–Streptomycin [18]. Human PSCs (CHA15 and iPS-NT4-S1) were kindly provided by CHA University (South Korea) and maintained as previously described [19]. Briefly, the cells were cultured under xeno-, serum-, and feeder-free conditions using E8 medium (STEMCELL Technologies, Vancouver, Canada) on dishes coated with recombinant human vitronectin (STEMCELL Technologies). They were subcultured at 70–80% confluency and passaged every 4–5 days by mechanical dissociation. All cells were maintained at 37 °C in a humidified atmosphere with 5% CO_2_.

### 2.2. Stepwise Differentiation of hPSCs into AECs

Multistep AEC differentiation was performed as previously described with some minor modifications [17]. Briefly, undifferentiated hPSCs were dissociated and then plated in dishes coated with vitronectin at a density of 1 × 10^5^ cells/cm^2^. After an overnight incubation, AEC differentiation was initiated with exposure to stepwise induction medium and was assessed by observing the expression of alveolar progenitors and AEC-specific markers on day 25 post-initiation using immunofluorescence staining and real-time quantitative PCR (qPCR).

### 2.3. Generation of Multicellular AOs from hPSCs

Generation of multicellular AOs was performed by combination of previously reported protocols with minor modifications [17,20,21]. Briefly, the AEC cultures were dissociated on day 14 of AEC differentiation with 0.4 U/mL collagenase B (Roche, Basel, Switzerland) for 2 h in a 37 °C incubator, followed by treatment with cell dissociation buffer (Gibco, Waltham, MA, USA) for 10 min in a 37 °C water bath to singularize cells. The single-cell suspension was then passed through a 70 μm cell strainer (BD Bioscience, Franklin Lakes, NJ, USA) and seeded into 96-well round-bottom plates (Corning, New York, NY USA, 5 × 10^4^ cells per well) containing AEC maturation medium supplemented with 10 μM ROCK inhibitor (STEMCELL Technologies). After distribution, 150 μL of 1:15 diluted Matrigel was added into each well to improve adhesion between cells. The plates were centrifuged at 450× *g* for 5 min and incubated overnight to allow aggregation at 37 °C in a humidified atmosphere with 5% CO_2_. After overnight culture, the aggregates were transferred to 6-well low-attachment plates (Corning) containing fresh AEC maturation medium and cultured for 11 days to establish AOs.

### 2.4. PM Preparation and Treatment

The PM2.5 (Diesel Particulate Matter, NIST, SRM^®^ 1650b) was purchased from Sigma-Aldrich Chemical Co. (St. Louis, MO, USA). A 10 mg/mL stock solution of dPM2.5 was prepared in dimethylsulfoxide (DMSO; Invitrogen-Gibco, Carlsbad, CA, USA) and stored at −4 °C until use. Immediately before the experiments, the stock solution was subjected to ultrasonification on ice for 10 min and subsequently diluted in culture medium to final concentrations. A549 cells were treated with dPM2.5 at concentrations of 0, 50, 100, and 200 μg/mL, respectively, for 48 h. dPM2.5 was added during AEC differentiation and AO development from hPSCs.

### 2.5. Cell Viability Detected Using Neutral Red Assay

Cell viability was measured by neutral red uptake assay as previously reported [22]. A549 cells were seeded on 96-well plates at a density of 1 × 10^4^ cells per well and placed in a humidified atmosphere at 37 °C with 5% CO_2_. After overnight culture, dPM2.5 was added to each well and the cells were incubated for 48 h. dPM2.5 treatment was replaced with neutral red solution (50 mg/mL in culture medium without FBS) for 3 h. Neutral red solution was aspirated and 1% formalin was added to each well for 1 min. After aspiration of fixative, the neutral red extracted solution (1% acetic acid in 50% ethanol) was added to each well, and the plate was placed on a microplate plate shaker for 10 min. Optical density value was tested at 540 nm by microplate reader (Bio-Tek, Winooski, VT, USA). Cell viability was calculated according to the manufacturer’s instructions.

### 2.6. Hematoxylin and Eosin and Immunofluorescence Staining

Paraffin sections of AOs were deparaffinized by sinking in xylene and sequentially rehydrated through a gradual concentration series, ending with deionized water. The sections were stained to nucleus using hematoxylin (BBC Biochemical, McKinney, TX, USA) and rinsed by water, followed by a short exposure to acidic ethanol. The sections also were exposed by eosin targeting to cytoplasm and dehydrated through a sequential concentration change, starting from 70% ethanol and increasing to 100% ethanol. After exposure to xylene three times, the sections were mounted with Permount mounting medium (Thermo Scientific, Waltham, MA, USA). For immunofluorescence staining, 4 μm thick AO sections were dewaxed with xylene and rehydrated with a gradient of ethanol. The sections were subjected to antigen retrieval with a citrate buffer bath (pH 6) at boiling temperature and then blocked for endogenous peroxidase activity. After washing with phosphate-buffered saline (PBS), the sections were incubated with primary antibodies against epithelial cell adhesion molecule (EPCAM; Santa Cruz Biotechnology, Dallas, TX, USA, sc-66020), carboxypeptidase M (CPM; Santa Cruz Biotechnology, sc-74380), homeodomain-only protein (HOPX; Santa Cruz Biotechnology, sc-398703), surfactant protein C (SFTPC; Abcam, ab40879), aquaporin 5 (AQP5; Thermo Scientific, PA5-36529), podoplanin (T1α; hermo Scientific, PA5-82301), and VIMENTIN (Santa Cruz Biotechnology, sc-373717) overnight at 4 °C. The sections were rinsed with PBS and incubated with secondary fluorescein isothiocyanate- or red fluorescent protein-labelled antibodies for 30 min. Finally, the sections were rinsed with PBS, counterstained with 4′,6-diamidino-2-phenylindole (DAPI) (Abcam, Cambridge, UK, ab104139), and observed under a fluorescence microscopy (IX-51, Olympus, Tokyo, Japan).

### 2.7. Western Blot Analysis

Protein extracted from hPSC-derived AECs were lysed in protein lysis buffer and quantified using the bicinchoninic acid protein assay. The 20 μg of protein was separated by SDS-PAGE using 8–15% gel and then transferred to polyvinylidene fluoride membranes. Nonspecific binding proteins were blocked with 5% skim milk for 1 h at room temperature. The membranes were incubated with primary antibodies against anti-phospho-p44/42 mitogen-activated protein kinase (Cell Signaling Technology, Danvers, USA, 4370), anti-p44/42 MAPK (Cell Signaling Technology, 4695), anti-SFTPC (Abcam, ab40879), anti-ACE2 (R&D, Minneapoli, MN, USA, AF933), and anti-fibronectin (FN) (Santa Cruz Biotechnology, sc-59826) overnight at 4 °C. Membranes were scanned with ChemiDoc imaging system (Bio-Rad Laboratories, Hercules, CA, USA).

### 2.8. RNA Extraction and Quantitative Real-Time qPCR

Total RNA was extracted from A549, AECs, AOs and undifferentiated hPSC cultures using an RNeasy Mini kit (Qiagen, Duesseldorf, Germany), and cDNA was synthesized using TOPscrip™ RT DryMIX (Enzynomics, Daejeon, Korea). PCR amplification was performed using a Step One Plus real-time PCR system (Applied Biosystems, Warrington, UK) with TOPreal™ qPCR 2X PreMIX (Enzynomics). Relative expression was normalized against glyceraldehyde 3-phosphate dehydrogenase (GAPDH) expression by the ΔΔCt method. The primer sequences are listed in Table 1.

### 2.9. Statistical Analysis

Values for all measurements are presented as the mean ± standard deviation (SD). Statistical significance was determined using Student’s *t*-test, with *p* < 0.05 considered statistically significant [21]. *p*-values are represented as *, <0.05, and as **, <0.01.

## 3. Results

### 3.1. dPM2.5 Disturbed hPSC Differentiation towards AECs

**Figure 1:**
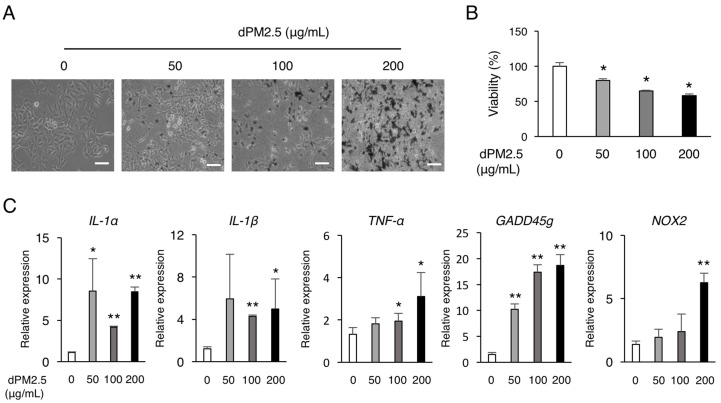
Diesel fine particulate matter (dPM2.5) decreased viability and induced inflammation. (**A**) Representative images of A549 cells cultured in various concentrations of dPM2.5 for 48 h. Bars, 100 μm. (**B**) Viability of the cultured human A549 cells exposed to 0–200 μg/mL dPM2.5 was determined by neutral red assay. The neutral red assay revealed that incubation with dPM2.5 decreased the viability in a concentration-dependent manner. (**C**) qPCR analysis for cell stress-related genes. Bars indicate the mean ± SD. * *p* < 0.05, ** *p* < 0.01.

**Figure 2:**
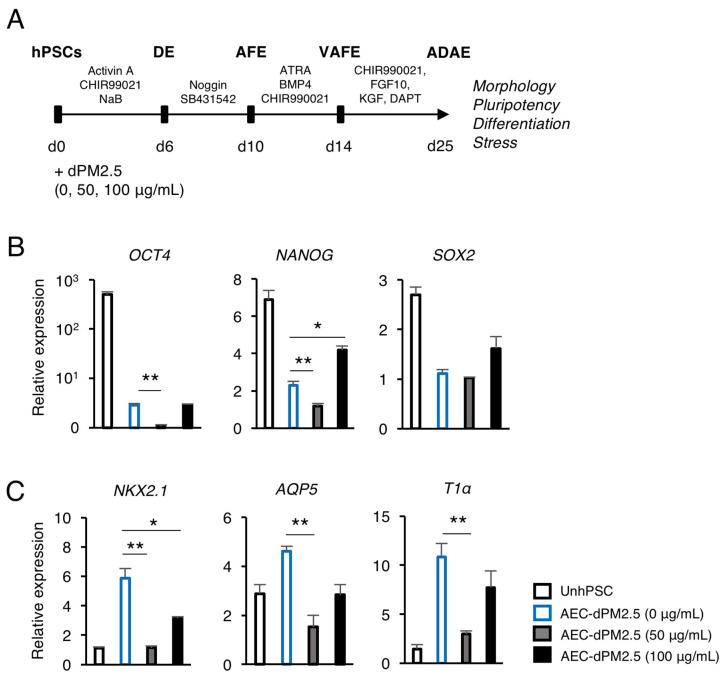
Effects of dPM2.5 exposure on hPSC-derived alveolar epithelial cell (AEC) differentiation. (**A**) Schematic diagram of stepwise AEC differentiation from hPSCs based on lung developmental process and dPM2.5 treatment. (**B**,**C**) qPCR analysis for the expression of pluripotency (*OCT4*, *NANOG*, and *SOX2*) and alveolar epithelium (*NKX2.1*, *AQP5*, and *T1α*) markers. Data is presented as mean ± SD. * *p* < 0.05, ** *p* < 0.01. UnhPSC, undifferentiated hPSC; DE, definitive endoderm; AFE, anterior foregut endoderm; VAFE, ventral anterior foregut endoderm; ADAE, alveolar and distal airway epithelium.

We first examined the cytotoxic effect of various concentrations of dPM2.5 (0, 50, 100, and 200 μg/mL) on A549 cell line. The cells in the dPM2.5 treatment groups exhibited elongated and fibroblast-like morphology (Figure 1A). The neutral red assay revealed that dPM2.5 exposure significantly reduced the viability of A549 cells in a dose-dependent manner (Figure 1B). Cell viability was decreased to 79.55 ± 2.45% by 50 μg/mL of dPM2.5, but showed greater decrease (57.95 ± 2.46%) at higher concentration of dPM2.5 (200 μg/mL). We also found that inflammation and apoptosis-related genes were significantly upregulated at low concentrations of dPM2.5 (50 and 100 μg/mL) (Figure 1C). On the basis of these results, we evaluated the potential developmental toxicity of dPM2.5 at concentrations of 50 and 100 μg/mL during hPSC-derived alveolar development. Using an optimized stepwise induction protocol, we induced undifferentiated hPSCs to AECs in the absence or presence of dPM2.5 and analyzed the expression patterns of pluripotency and alveolar lineage markers (Figure 2A). qPCR analysis revealed downregulation of pluripotency genes (*OCT4*, *NANOG*, and *SOX2*) and upregulation of alveolar epithelium genes (*NKX2.1*, *AQP5*, and *T1α*), indicating that our AEC differentiation process was efficient (Figure 2B,C). Notably, exposure of dPM2.5 during AEC differentiation markedly reduced the expression of the alveolar epithelium genes (Figure 2C). These results suggest that dPM2.5 exposure may interfere with the development of the human alveolar epithelium.

### 3.2. dPM2.5 Promoted EMT during hPSC-Derived AEC Differentiation via Activation of ERK Signaling

**Figure 3:**
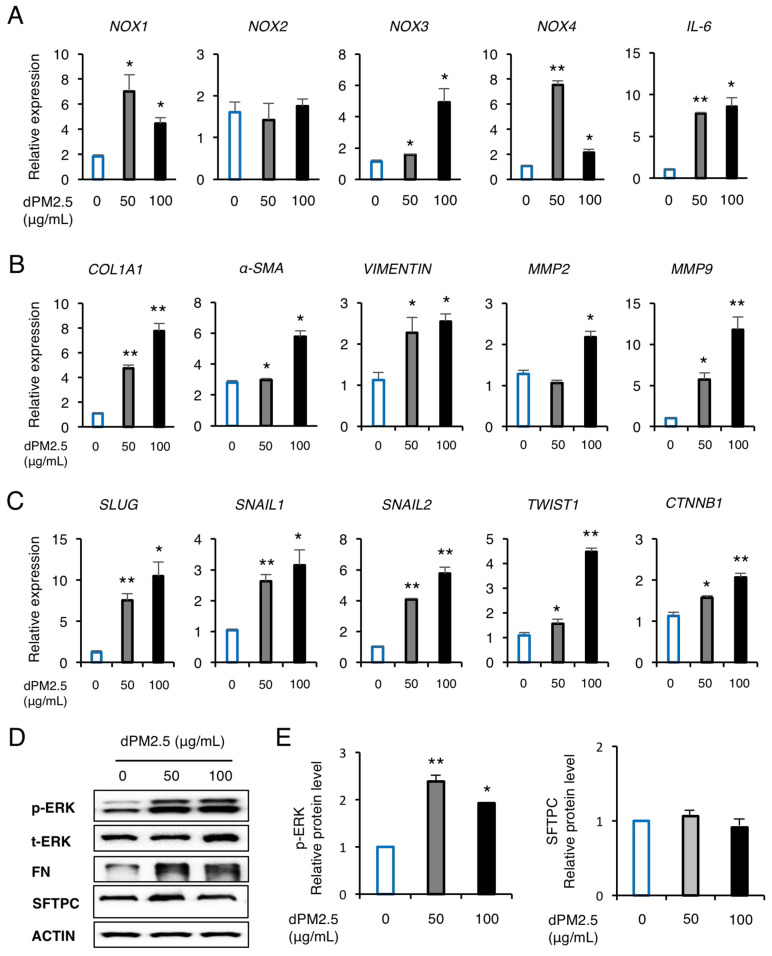
dPM2.5 induced epithelial-to-mesenchymal transition (EMT) during hPSC-derived AEC differentiation. (**A**–**C**) Transcript levels of inflammation- (**A**), fibrosis- (**B**), and EMT-related (**C**) genes were measured using qPCR. (**D**,**E**) Western blotting and subsequent quantification of p-ERK and SFTPC in hPSC-AECs cultured in the absence and presence of dPM2.5. ACTIN was used as a loading control. Data are presented as mean ± SD. * *p* < 0.05, ** *p* < 0.01 (vs. 0 μg/mL control).

We next investigated the expression of inflammation, oxidative stress, and EMT-related genes by dPM2.5 exposure during hPSC-derived AEC differentiation. We observed significant upregulation of pro-inflammatory interleukin-6 (*IL-6*) cytokine by treatment with dPM2.5 (50 and 100 μg/mL), which may be related with upregulation of nicotinamide adenine dinucleotide phosphate (NADPH) oxidases (*NOX*-*1*, *3*, and *4*) known as major sources of reactive oxygen species (ROS) (Figure 3A). We also found that exposure to dPM2.5 markedly increased the transcript levels of the mesenchymal markers including matrix metalloproteinase (*MMP*)-2, *MMP-9*, *COL1A1*, *α-SMA*, and *VIMENTIN* (Figure 3B). To further investigate the mechanism of how dPM2.5 promotes EMT, we measured the expression levels of EMT-related downstream signaling and transcriptional factor (TF) cluster. We found that exposure to dPM2.5 upregulated the expression of EMT-mediated nuclear TFs, such as *SLUG*, *SNAIL1/2*, *TWIST*, and catenin beta 1 (*CTNNB1*) in a dose-dependent manner (Figure 3C). In addition, western blot analysis revealed that dPM2.5 (50 μg/mL) significantly increased the phosphorylation of extracellular signal-regulated kinase (ERK) and FN (Figure 3D). These results indicated that dPM2.5-induced cellular and molecular alterations such as EMT and oxidative stress might be involved in disturbing hPSC differentiation towards AECs. AECs are functionally characterized by the production of hydrophobic SFTPC, which forms a thin surface layer that covers the AEC surface. SFTPC plays a key role in preventing alveolar collapse by reduction of surface tension and recovering from lung injury due to toxic materials [23]. Thus, we examined the effect of dPM2.5 exposure on expression of SFTPC during hPSC-derived AEC differentiation. Western blot analysis showed that SFTPC expression was not affected by treatment of dPM2.5 (50 and 100 μg/mL) (Figure 3D). These results suggest that hPSC-derived AECs may have a protective mechanism against dPM2.5 cytotoxicity via secretion of surfactant proteins.

### 3.3. dPM2.5 Induced EMT during 3D Alveolar Development

**Figure 4:**
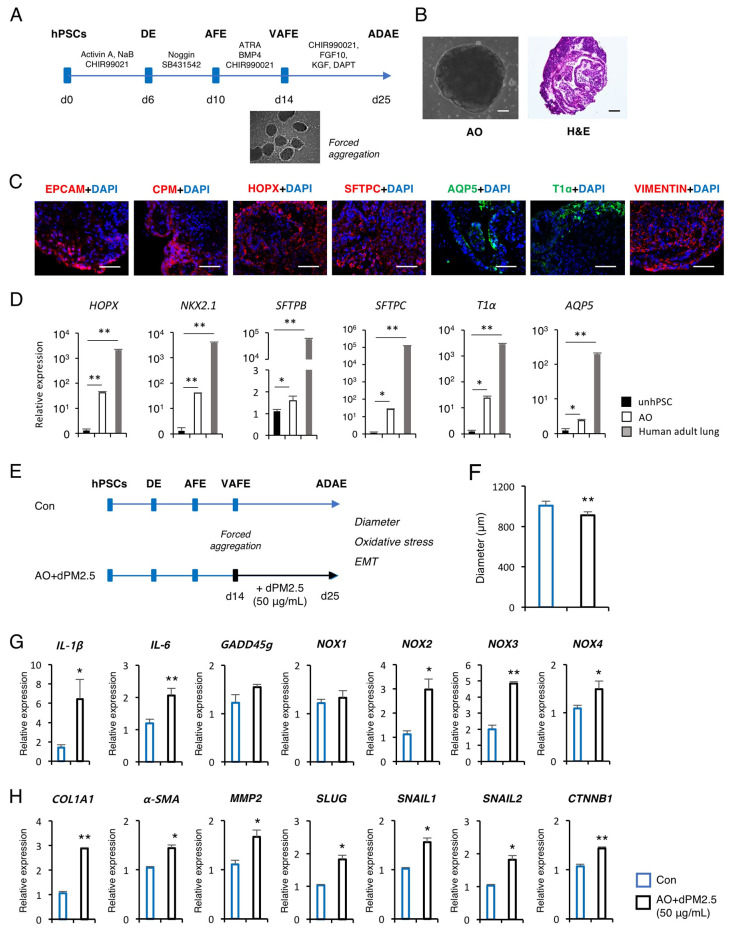
Effects of dPM2.5 exposure during hPSC-derived alveolar organoid (AO) development. (**A**) Schematic diagram of AO generation from hPSCs. (**B**) Representative bright field and H&E staining images of AOs. Scale bars, 100 μm. (**C**) Immunofluorescence staining for AEP (EPCAM, CPM, and HOPX, red), AEC1 (AQP5 and *T1α*, green), AEC2 (SFTPC, red) and mesenchymal stromal cell (VIMENTIN, red) markers in AOs on day 25 of induction. Nuclei were counterstained with 4′,6-diamidino-2-phenylindole (DAPI) (blue). Scale bars, 100 μm. (**D**) qPCR analysis of the indicated AEP and AEC markers in AOs. Data are shown as fold-change relative to undifferentiated hPSCs (unhPSC). Data is presented as mean ± SD. * *p* < 0.05, ** *p* < 0.01. (**E**) Schematic diagram of dPM2.5 treatment during hPSC-derived AO development. Black lines indicate the duration of dPM2.5 treatment. Scale bars, 100 μm. (**F**) Measurement of AO size was taken by averaging the longest and shortest diameter of AOs. (**G**,**H**) Transcript levels of inflammation, fibrosis, and EMT-related genes were measured using qPCR. Data is presented as mean ± SD. * *p* < 0.05, ** *p* < 0.01 (vs. Control).

Next, we investigated the developmental toxicity of dPM2.5 using in vitro human AO model generated from hPSCs. Previously, we and others have reported efficient and reproducible protocols for the production of functional AECs from hPSCs and formation of cellular aggregates from a single-cell suspension of hPSCs by forced aggregation [17,20] (Figure 4A). By taking advantage of these protocols, we generated uniform aggregates and cultured under AEC maturation medium for 11 days to establish AOs, which exhibited an alveolar sac-like structure with multiple alveoli and layers of epithelial cells (Figure 4B). Immunofluorescence staining showed that AOs express markers of alveolar epithelial progenitor (AEP) (EPCAM and CPM), type 1 AEC (AEC1; AQP5 and T1α), type 2 AEC (AEC2; SFTPC), and mesenchymal stromal cells (VIMENTIN) (Figure 4C). Moreover, these markers were robustly expressed in AOs compared with undifferentiated hPSC cultures, but shown to have lower transcript levels compared with human adult lung tissue (Figure 4D). These observations confirmed that the AO is a construct that contains multiple cell types of alveolar tissue and has a fetal level of maturation.

We employed the in vitro 3D AO induction system to investigate the potential adverse effects of dPM2.5 on the prenatal stage of alveolar development. At 14 days of differentiation, we treated the forced aggregates with dPM2.5 (50 μg/mL) and cultured under AEC maturation medium for up to 25 days (Figure 4E). dPM2.5-treated AOs showed a significant decrease in diameters compared with the control (Figure 4F). We observed significant upregulation of pro-inflammatory cytokines (*IL-1β* and *IL-6*) and NOXs (*NOX-2, 3* and *4*) by treatment with dPM2.5 (Figure 4G). We also found that exposure to dPM2.5 markedly increased the transcript levels of the mesenchymal markers including *COL1A1*, *α-SMA*, and *MMP-2*, which are associated with upregulation of EMT-mediated nuclear TFs including *SLUG*, *SNAIL1/2*, and *CTNNB1* (Figure 4H). Taken together, these data suggest that exposure to dPM2.5 during 3D alveolar development induces fibrotic changes by triggering inflammation and EMT.

### 3.4. dPM2.5 Enhanced ACE2 and TMPRSS2 Expression in hPSC-Derived AECs and AOs

**Figure 5:**
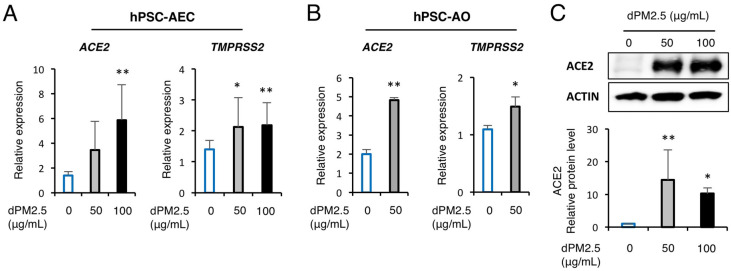
dPM2.5 enhanced *AEC2* and *TMPRSS2* expression in hPSC-derived AECs and AOs. (**A**,**B**) Transcript levels of *ACE2* and *TMPRSS2* were measured using qPCR in hPSC-derived AECs (**A**) and AOs (**B**). Data are presented as mean ± SD. * *p* < 0.05, ** *p* < 0.01. (**C**) Western blotting and subsequent quantification of ACE2 in hPSC-AECs cultured in the absence and presence of dPM2.5. ACTIN was used as a loading control. Data are presented as mean ± SD. * *p* < 0.05, ** *p* < 0.01 (vs. 0 μg/mL control).

Since new coronavirus disease (COVID-19) caused by SARS-CoV-2 was first reported in China, recent studies proposed a potential relationship between PM2.5 concentrations and the severity of SARS-CoV-2, which might be closely associated with altered expression of ACE2, the entry receptor for SARS-CoV-2, by chronic exposure to PM2.5. Thus, we further investigated the effect of dPM2.5 exposure on expression of AEC2 and its cofactor TMPRSS2 during 2D and 3D alveolar development. Notably, dPM2.5 (50 and 100 μg/mL) significantly increased ACE2 and TMPRSS2 expression in hPSC-derived AECs (Figure 5A). We also found that exposure to dPM2.5 during 3D AO development significantly increased the transcript levels of ACE2 and TMPRSS2 (Figure 5B). In addition, western blot analysis revealed that ACE2 protein expression in hPSC-derived AECs was significantly increased following exposure to dPM2.5 (50 and 100 μg/mL) (Figure 5C). There findings suggest that exposure to dPM2.5 may facilitate the interaction between the spike protein of SARS-Cov-2 and ACE2 by upregulation of AEC2 and TMPRSS2 in alveolar tissue.

## 4. Discussion

Growing epidemiological investigations link prenatal exposure to fine particulate matter (PM2.5) with reduced lung function and incidence of pulmonary diseases in infancy and childhood [2,3,4,5,6,7,8]. However, cellular and molecular evidence regarding the effects of prenatal PM2.5 exposure on the development of human fetal lung is insufficient due to rare accessibility to primary human tissues and lack of a biologically relevant in vitro human lung development model. Although human primary AECs (hpAECs) and fetal lung fibroblasts are the most appropriate sources for evaluating the cytotoxicity of dPM2.5, primary cells derived from different donors can show distinct responses depending on genetic background, patient age, and the type of tissue source [24]. Furthermore, their phenotypic and functional properties may change over time of in vitro culture. In the present study, we utilized, for the first time, an hPSC-based 2D and 3D alveolar differentiation system for evaluating in vitro developmental toxicity of dPM2.5. Our system is based on optimized stepwise direct hPSC differentiation via mimicking of early alveolar developmental cues in a temporally controlled manner [25]. Furthermore, we have previously demonstrated that hPSC-derived AECs displayed similar phenotypes and cellular responses to cadmium exposure as those of hpAECs [26]. Using our efficient and reproducible protocols, we found that dPM2.5 exposure perturbed AEC differentiation and resulted in loss of epithelial features of hPSC-derived AECs and AOs, which was evidenced by upregulation of mesenchymal-related genes and EMT-mediated nuclear TFs. More interestingly, dPM2.5 treatment during hPSC-derived AEC and AO development triggered a marked upregulation of SARS-CoV-2 receptor ACE2 and its cofactor TMPRSS2 (Figure 5). This study suggests that our optimized hPSC-based alveolar differentiation system could be a biologically relevant in vitro alternative for recapitulating the phenotypes and functions of in vivo fetal alveolar tissues and understanding key cellular and molecular features of prenatal PM2.5 toxicity.

We demonstrated that AECs and AOs exposed to dPM2.5 exhibited fibrotic features, accompanied by upregulation of mesenchymal markers (*COL1A1*, *α-SMA*, and *VIMENTIN)* and NOX genes, which were promoted by activation of TGF-β1-mediated ERK signaling and EMT-related TFs. Although epidemiological studies providing linkages between prenatal PM2.5 exposure and the EMT progress of fetal lung have not been published, experimental evidences from animal and human airway epithelial cell lines suggest that PM2.5 contributes to EMT of alveolar epithelium through TGF-β1-mediated activation of mothers against decapentaplegic (SMAD) or ERK signaling pathway [27,28,29]. Recently, Lee et al. [30] reported that dPM2.5 promotes EMT of human retinal epithelial cells via TGF-β1-mediated activation of SMAD/ERK signaling pathway, suggesting that dPM2.5–ERK–EMT signaling might be a conserved regulatory axis in fibrotic changes of epithelial cells of other organs such as kidney and liver.

Recent epidemiological investigations proposed a potential relationship between PM2.5 concentrations and the susceptibility severity of SARS-CoV-2 [31,32,33,34], which might be closely associated with altered expression of ACE2 by chronic exposure to fine PM2.5 [35]. Recently, computational analysis of sequencing data from patients with chronic lung diseases revealed that dysregulation of androgen, hypoxia-inducible factor 1-alpha, interferon, and IL-6 cytokine pathways is associated with upregulation of ACE2 and SARS-Cov-2 severity [36,37,38,39,40]. To the best of our knowledge, this is the first study reporting the transcriptional and translational upregulation of ACE2 in hPSC-derived AECs and 3D AOs by exposure to dPM2.5. ACE2 protects against renin–angiotensin system-induced lung injuries by cleaving Angiotensin II (Ang II) to limit substrate availability in the adverse AEC–Ang II–Ang II receptor 1 axis and generating Ang 1–7, which act through Mas receptors [41]. Animal studies demonstrated that ACE2 knockout mice displayed more severe symptoms of acid-induced acute lung injury compared with wild-type control and are more prone to develop acute pulmonary inflammation after exposure to PM2.5, suggesting a protective role of increased pulmonary ACE2 in lung injuries from environmental toxins [42,43]. In addition, Takano et al. [44], hypothesized that surfactant proteins may be a strong defender against SARS-Cov-2, suggesting that inhalation of chemical surfactants or surfactant production stimulants may be effective for the treatment of COVID-19. Therefore, these findings suggest that robust expression of ACE2 and SFTPC in hPSC-AECs and AOs may have a protective role against dPM2.5 cytotoxicity, but on the other hand increase a risk of SARS-CoV-2 infection.

Recent studies suggest that 3D configurations, such as organoids and spheroids, take clear advantages over conventional 2D monolayer cultures for more faithfully recapitulating the complexity and functions of in vivo tissues [13]. Here, using hPSC-derived 3D AOs, we provided the first report investigating the adverse effects of dPM2.5 exposure during alveolar development. The AOs contains multiple cell types of alveolar tissue and phenotypically resemble in vivo alveolar tissue. Importantly, the AOs showed a lower expression level of alveolar epithelium markers compared with that of adult lung tissue, indicating a fetal level of maturation. A recent animal study demonstrated that maternal exposure to PM2.5 reduced distal alveolar epithelial-related genes and disturbed the distal epithelium differentiation at embryonic day 18.5 (E18.5), which is the saccular stage emerging clusters of epithelial sacs that will later develop into alveoli [45]. However, maternal exposure to PM2.5 had no impact on earlier lung developmental stages (E0–E16.5). These findings suggest that our AOs offer an appropriate in vitro model to evaluate the effects of prenatal exposure to PM2.5 on fetal alveolar development. Although AOs used in this study contain multiple alveolar cell types, the AOs remain incomplete as they lack vasculature and tissue resident immune cells, which create a critical microenvironment relevant for developmental and pathological processes. Thus, development of AO closer to the native tissue architecture and function by incorporation of essential components such as endothelial cells and alveolar macrophages is required to provide a more robust in vitro model for evaluating developmental toxicity of environmental toxins.

## 5. Conclusions

Taken together, the current study suggested that our optimized hPSC-based 3D alveolar differentiation system may serve as a relevant in vitro model to recapitulate the structure and functions of in vivo fetal alveolar tissues and understand key cellular and molecular features of prenatal PM2.5 toxicity and virus biology. Furthermore, our study provides the first evidence that dPM2.5 stimulates AEC2 and TMRPSS2 expression in human AECs and AOs derived from hPSCs, providing the biological plausibility for epidemiological studies reporting the relationship between PM2.5 concentrations and the severity of COVID-19 disease.

## Figures and Tables

**Table 1:** Primer sequences used for qPCR.

Genes		Sequence 5′ to 3′	Product Size (bp)
*T1α*	F	TGC GAA AAA TGT CGG GAA GG	51
	R	GGC GTA ACC CTT CAG CTC TT	
*SFTPB*	F	GCC ATA CCA CAG GCA ATG CT	80
	R	TGC TGC TCC ACA AAT TGC TT	
*SFTPC*	F	CCT TCT TAT CGT GGT GGT GGT	96
	R	TCT CCG TGT GTT TCT GGC TCA T	
*HOPX*	F	GCC TTT CCG AGG AGG AGA C	97
	R	TCT GTG ACG GAT CTG CAC TC	
*AQP5*	F	ACT GGG TTT TCT GGG TAG GG	172
	R	ATG GTC TTC TTC CGC TCT TC	
*NKX2.1*	F	AGC ACA CGA CTC CGT TCT CA	75
	R	CCT CCA TGC CCA CTT TCT TG	
*VIMENTIN*	F	CCA GGC AAA GCA GGA GTC	212
	R	CGA AGG TGA CGA GCC ATT	
*α-SMA*	F	GAC GAA GCA CAG AGC AAA AG	70
	R	AGT TGG TGA TGA TGC CAT GT	
*COL1A1*	F	AAG GGT GAG ACA GGC GAA CA	70
	R	GAC CCT GGA GGC CAG AGA AG	
*CTNNB1*	F	AAA ATG GCA GTG CGT TTA	99
	R	TTT GAA GGC AGT CTG TCG TA	
*TWIST1*	F	AGC AAG ATT CAG ACC CTC AAG	145
	R	ATC CTC CAG ACC GAG AAG G	
*SNAIL1*	F	TTT ACC TTC CAG CAG CCC TA	73
	R	GAC AGA GTC CCA GAT GAG CA	
*SNAIL2*	F	CTG TGG GGA CAT GAA CTG TG	115
	R	AGG GTC TGG GGA AAC TCG	
*IL-1α*	F	CCA ACG GGA AGG TTC TGA AG	70
	R	GCC TCC AGG TCA TCA TCA GT	
*IL-1β*	F	CTG TCC TGC GTG TTG AAA GA	179
	R	TTC TGC TTG AGA GGT GCT GA	
*IL-6*	F	AGC CCT GAG AAA GGA GAC AT	85
	R	TGG AAG GTT CAG GTT GTT TT	
*TNF-α*	F	AAC CTC CTC TCT GCC ATC AA	185
	R	CCA AAG TAG ACC TGC CCA GA	
*NOX1*	F	AGG GCT TTC GAA CAA CAA TA	104
	R	CCA GCA CAG CCA CTT CAT AC	
*NOX2*	F	AAC TGC TGG AGA GCC AGA TG	101
	R	GCA AAG TGA TTG GCC TGA GA	
*NOX3*	F	GCT ATG CAG AAT GGC AGA CA	101
	R	TAC AAG ACC ACA GGG CCT AA	
*NOX4*	F	CTT TTG GAA GTC CAT TTG AG	231
	R	GTC TGT TCT CTT GCC AAA AC	
*OCT4*	F	TCG AGA ACC GAG TGA GAG G	125
	R	GAA CCA CAC TCG GAC CAC A	
*SOX2*	F	GCA CAT GAA GGA GCA CCC GGA TTA	86
	R	GTG GTC CTT CTT GTG CTG C	
*NANOG*	F	CAA AGG CAA ACA ACC CAC TT	158
	R	TCT GCT GGA GGC TGA GGT AT	
*GADD45g*	F	CAG ATC CAT TTT ACG CTG ATC CA	209
	R	TCC TCG CAA AAC AGG CTG AG	
*SLUG*	F	TGT GAC AAG GAA TAT GTG AGC C	203
	R	TGA GCC CTC AGA TTT GAC CTG	
*MMP2*	F	AGC GAG TGG ATG CCG CCT TTA A	138
	R	CAT TCC AGG CAT CTG CGA TGA G	
*MMP9*	F	CTC GAA CTT TGA CAG CGA CA	187
	R	GCC ATT CAC GTC GTC CTT AT	
*ACE2*	F	GGGATCAGAGATCGGAAGAAGAAA	124
	R	AGGAGGTCTGAACATCATCAGTG	
*TMPRSS2*	F	AATCGGTGTGTTCGCCTCTAC	106
	R	CGTAGTTCTCGTTCCAGTCGT	
*GAPDH*	F	GGC ATG GAC TGT GGT CAT GA	87
	R	TGC ACC ACC AAC TGC TTA GC	
